# Using The Cancer Genome Atlas data to refine the 8th edition of the American Joint Committee on Cancer staging for papillary thyroid carcinoma

**DOI:** 10.1007/s12020-020-02434-x

**Published:** 2020-09-11

**Authors:** Anello Marcello Poma, Elisabetta Macerola, Liborio Torregrossa, Rossella Elisei, Ferruccio Santini, Fulvio Basolo

**Affiliations:** 1 Department of Surgical, Medical, Molecular Pathology and Critical Area, University of Pisa, Via Savi 10, 56126 Pisa, Italy; 2 Department of Laboratory Medicine, Section of Pathology, University Hospital of Pisa, Via Roma 57, 56126 Pisa, Italy; 3 Department of Clinical and Experimental Medicine, Unit of Endocrinology, University of Pisa, Via Paradisa 2, 56124 Pisa, Italy

**Keywords:** AJCC staging, Papillary Thyroid Carcinoma, PTC, TCGA data, Prognosis, Outcome

## Abstract

**Purpose:**

The 8th edition of the American Joint Committee on Cancer (AJCC) staging led to a significant downstaging of well differentiated thyroid cancer patients. However, some patients who had been downstaged still experienced death. By using data from the thyroid cancer dataset of The Cancer Genome Atlas (TCGA), we aimed to find molecular features that could improve survival prediction.

**Methods:**

TCGA data were downloaded from cBioPortal. Restaging of cases was performed according to the pathological reports.

**Results:**

Out of 496 cases, 204 (41.1%) were downstaged, and the proportion of deaths increased in stages III and IV. *TERT* promoter mutations were no longer enriched in stage IV only, but significantly redistributed also in stages II and III. *TERT* mutation was the only alteration predictive of poor survival; however, in this series it was not independent from the AJCC staging. Five proteins (4E-BP1_pT70, Chk1_pS345, Snail, STAT5 alpha and PAI-1) were significantly associated with survival, and their use as a panel refined the risk stratification independently from the AJCC staging, with a hazard ratio for a positive result of 21.2 (95%CI 3.7–122.2, *P* = 0.0006).

**Conclusions:**

In the TCGA series, the proportion of deaths is in line with the expected survival of the latest AJCC staging, with a neat separation of risk among stages. Nevertheless, the use of protein expression can be useful in refining the stratification. Finally, after the restaging, a considerable number of tumors with *TERT* mutations will be allocated in lower stages; hence, dedicated studies should define the prognostic usefulness of these mutations in low-stage diseases.

## Introduction

The American Joint Committee on Cancer (AJCC) tumor-node-metastasis staging is the most effective and used stratification system designed to predict survival of patients. The 8th edition of the AJCC staging for thyroid cancer was released at the end of 2016 but is effective from the beginning of 2018. With respect to the 7th edition, the main changes for well differentiated thyroid cancer (WDTC) were: (i) the increase of the age cutoff from 45 to 55 years, (ii) the redefinition of T3 disease, which now does not encompass minimal extrathyroidal extension (ETE) detected only on histological examination, and (iii) the downgrading of positive lymph nodes to stage II for older patients [1, 2]. The consequent massive downstaging was intentional, due to the overall excellent survival of patients with WDTC, especially the youngest ones. The downstaging affected both stages III and IV that comprise now less than 5% of patients with WDTC. On the contrary, the amount of patients at stages I or II raised considerably [3–5]. Although the proportion of deaths in the lower stages decreased due to the overall increment of patients in those stages, a non-negligible number of patients who had been downstaged experienced death. Since the staging has many implications in the management of patients including treatment and follow-up, many authors have been questioning whether the current edition of the AJCC can be improved to guarantee a safer approach to a subgroup of patients [3, 6–8]. In particular, it was suggested that no sufficient consideration is given to the involvement of lateral lymph nodes [6, 8], T3b-T4 disease especially in patients aged 45–55 years [3, 6] and the presence of distant metastasis at diagnosis [3]. Mutations in *BRAF* and *TERT* have also been associated with disease specific mortality in papillary thyroid cancer (PTC) [9]; however, their role as independent predictor of survival is debated, and currently none of the validated risk systems includes molecular testing. By using clinical and molecular data from the thyroid cancer dataset of The Cancer Genome Atlas (TCGA) [10], we sought to identify potential features that could refine the stratification of patients independently from the AJCC staging.

## Materials and methods

### Acquisition of data and restaging

Mutation Annotation Format (MAF) file, clinical data, and level 3 reverse phase protein lysate microarray (rppa) data of the PTC dataset of the TCGA [10] were downloaded from cBioPortal (https://www.cbioportal.org/) [11, 12]. Clinical variables considered were histological type, age, gender, pN, pM, AJCC stage, American Thyroid Association (ATA) risk-group, distant metastasis, patient age, completeness of resection, local invasion, and tumor size (MACIS) score, patient’s vital status (as provided in the original data), persistence of disease, ETE, thyroid differentiation score (TDS), ERK score and number of nonsilent mutations. The restaging of cases was performed following the pathological report available on cBioPortal. The redistribution of cases between the 7th and the 8th edition of the AJCC staging was plotted using ggalluvial v.0.11.1 R package.

Within the MAF file, synonymous variants were ignored. Moreover, gene fusions and *TERT* promoter mutations that were available in separate files were manually added.

### Gene mutations enrichment

The most frequent alterations were plotted using maftools v.2.2.10 Bioconductor package [13]. The enrichment of gene mutations in specific clinical features was performed by a Fisher exact text with maftools package. In details, for features with two classes the pairwise false discovery rate (FDR) was considered, whereas for features with more than two classes the groupwise FDR was taken into account. FDR below 0.05 was considered significant.

### Receiver operating characteristic (ROC) analysis

For features presented as numeric variables (i.e., MACIS score, TDS, ERK score and number of nonsilent mutations) a ROC analysis was performed. Specifically, the Youden J statistic was used to select the best cutoff in discriminating patient’s vital status by the pROC v.1.15.3 R package [14]. Patients were then dichotomized according to the selected cutoffs.

As regards rppa expression data, an exploratory ROC analysis was performed following the procedures of the caret v.6.0–84 R package [15]. Proteins with an area under the curve (AUC) greater than 0.75 were selected for further analyses. Also for proteins, the best cutoff was assessed by the Youden J statistic.

### Survival analyses

Survival curves were estimated by the Kaplan–Meier method, and differences among curves were tested by log-rank test using the survival v.3.1–8 R package [16]. Survival curves were plotted using survminer v.0.4.6 R package. Multivariate analysis and estimation of hazard ratio (HR) were performed by Cox regression following the procedures of survival R package. *P* value below 0.05 was considered significant. All analyses were performed in R environment (v.3.6.1, https://www.r-project.org/).

## Results

### Restaging of cases from 7th to 8th edition of the AJCC

**Fig. 1:**
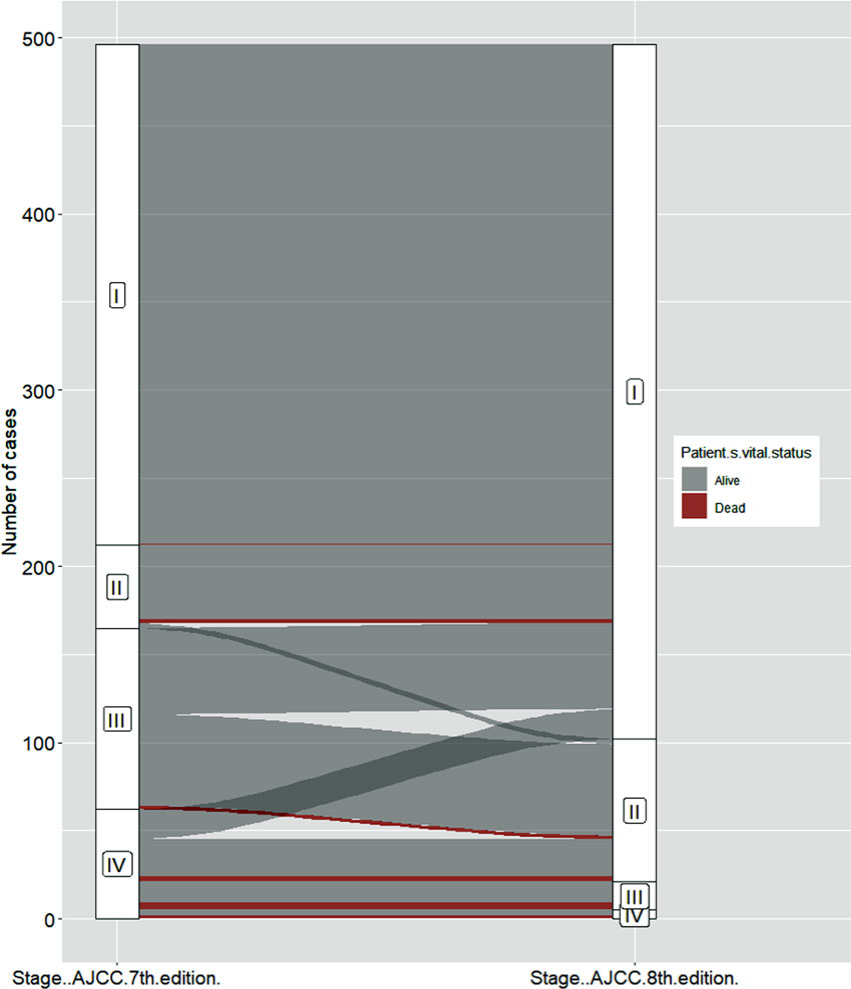
Alluvial plot. Redistribution of cases after the restaging. Adverse events are highlighted in red

Out of the 496 cases considered, 292 (58.9%) were confirmed in the same stage: 284 in stage I, three in stage II and five in stage IV, whereas 204 (41.1%) cases were downstaged. Details are reported in Table 1 and Fig. 1. The median follow-up was 14.3 months (IQ range 7.1–28.5 months). Considering the AJCC 7th edition staging, the proportion of deaths were 0.4% in stage I, 4.3% in stage II, 1.9% in stage III, and 14.4% in stage IV. After the restaging, the proportion of deaths were 0.7, 6.2, 25, and 40% for stages I, II, III, and IV, respectively.

**Table 1:** Restaging of cases according to the VIII edition of the AJCC

Cases confirmed in the same stage (*n* = 292)		
Stage	# of cases	adverse events
I	284	1
II	3	0
III	0	0
IV	5	2

Cases downstaged (*n* = 204)			
Stage 7th edition	Stage 8th edition	# of cases	adverse events
II	I	44	2
III	II	54	2
III	I	49	0
IV	III	16	4
IV	II	24	3
IV	I	17	0

### Enrichment of gene mutations in specific clinical features

**Fig. 2:**
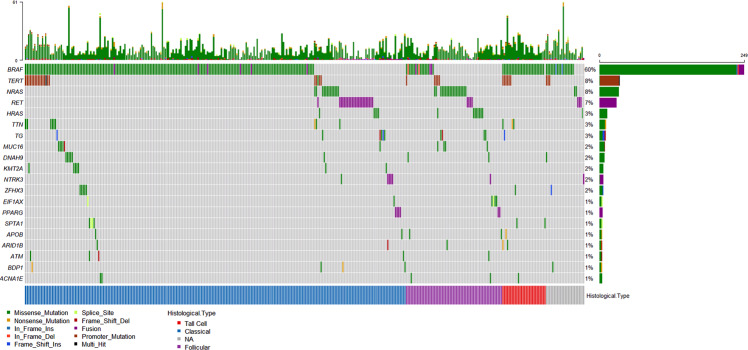
Oncoplot. The 20 most frequently mutated genes including fusions and *TERT* promoter mutation are reported. In the upper part of the plot, the number of nonsilent mutation is showed

The 20 most frequent nonsilent gene alterations including fusions and *TERT* promoter mutations were reported in Fig. 2.

*BRAF* mutations were enriched in classical variant (FDR < 0.0001) and tall cell variant (TC) (FDR = 0.0007) PTC, whereas *NRAS* and *HRAS* were associated with follicular variant (FV) (FDR < 0.0001 and FDR = 0.0292 respectively). Moreover, *NRAS* mutations were associated with pN0 (FDR = 0.002) and ATA low-risk (FDR = 0.0077); *BRAF* was enriched in pN1a (FDR = 0.002), minimal ETE (FDR < 0.0001) and ATA intermediate-risk (FDR = 0.0001), whereas *TERT* promoter mutations were overrepresented in cases with gross ETE (FDR = 0.0018), pM1 (FDR = 0.0086) and ATA high-risk (FDR < 0.0001). In addition, *TERT* mutations were enriched in patients with more than 45 years (FDR = 0.006), and even strongly in those with more than 55 years (FDR < 0.0001). As regards the AJCC staging, in the 7th edition *TERT* was overrepresented only in stage IV (FDR < 0.0001), whereas in the 8th edition it was enriched in stages IV (FDR = 0.0061), III (FDR = 0.0049) and II (FDR = 0.0119). Finally, *TERT* was the only gene associated with persistence of disease (FDR = 0.0025) and death (FDR = 0.0027). Results are summarized in Table 2.

**Table 2:** Enrichment of gene mutations in specific clinical features

Gene alteration	*RAS*^a^	*BRAF*	*TERT* promoter
Histological variant	FVPTC	CVPTC TCPTC	–
Lymph node status	pN0	pN1a	–
Extrathyroidal extension	–	Minimal	Gross
Distant metastasis	–	–	pM1
Age	–	–	>45 years >55 years
ATA risk	Low	Intermediate	High
AJCC 7th edition	–	–	Stage IV
AJCC 8th edition	–	–	Stages II, III, IV
Persistence of disease	–	–	Yes
Death	–	–	Yes

Only significant results are showed

^a^For histological variant, it refers to *HRAS* and *NRAS*; for the other features, it refers to *NRAS* only

*FVPTC* Follicular Variant of Papillary Thyroid Carcinoma, *CVPTC* Classical Variant of Papillary Thyroid Carcinoma, *TCPTC* Tall Cell Variant of Papillary Thyroid Carcinoma, *ATA* American Thyroid Association, *AJCC* American Joint Committee on Cancer

### Univariate and multivariate survival analyses

**Fig. 3:**
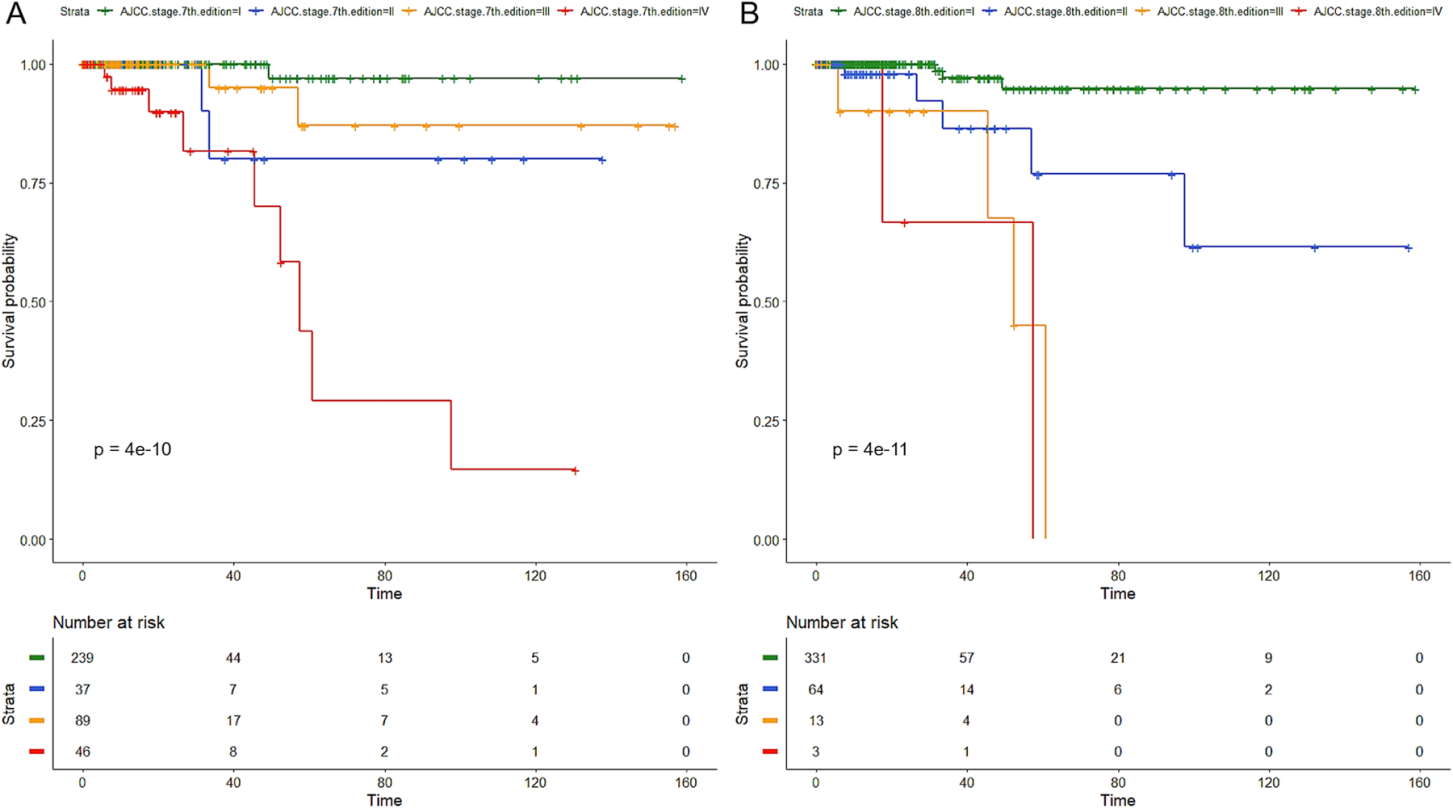
Kaplan–Meier curves according to the 7th (**a**) and 8th (**b**) edition of the AJCC staging. *P* refers to univariate *P* value. Time is expressed in months

The 8th edition of the AJCC staging produced a neater separation of curves than the 7th edition and a lower *P* value (Fig. 3). Among the other tested predictors, several were significant by univariate analysis including ATA risk stratification (*P* = 0.0008), ETE (*P* < 0.0001), age (*P* < 0.0001), MACIS score (*P* < 0.0001), TDS (*P* = 0.02), ERK score (*P* = 0.04), number of nonsilent mutations (*P* = 0.002) and *TERT* promoter mutations, both alone (*P* < 0.0001) or coexisting with *BRAF* or *RAS* (*P* < 0.0001). However, when fitting a multivariate Cox regression with the 8th edition of the AJCC, all the above-mentioned predictors were not independent.

### Rppa data analysis

**Fig. 4:**
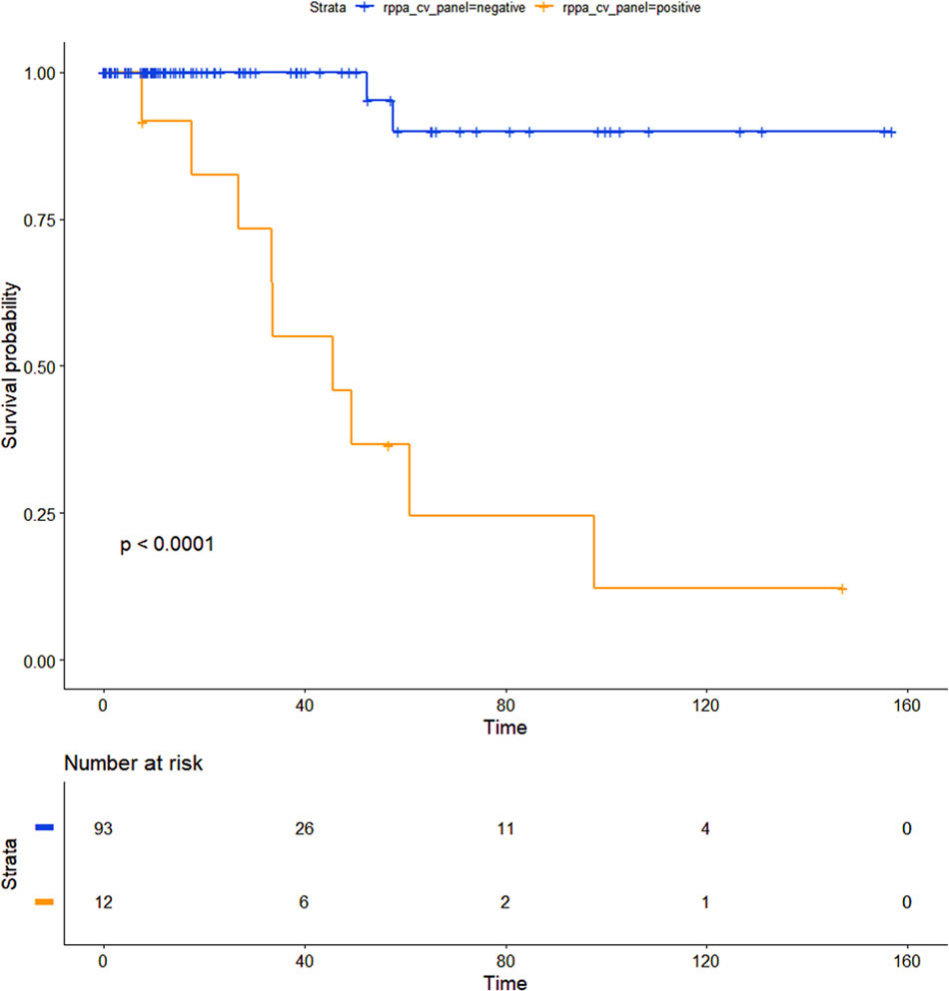
Kaplan–Meier curve obtained stratifying patients according to the five-protein panel. In detail, a positive result was assigned whenever at least four out of five markers were above (or below) the cutoff. P refers to univariate P value. Time is expressed in months

Since in the cases with rppa data (*n* = 222) the majority of adverse events were in CVPTC, and no event occurred in FVPTC and TCPTC, only CVPTC (*n* = 143) were considered for the following analyses. The exploratory ROC analysis revealed five proteins (4E-BP1_pT70, Chk1_pS345, Snail, STAT5 alpha and PAI-1) with an AUC greater than 0.75 in discriminating patient’s vital status. For each of these proteins, the expression value with the best Youden index was selected as cutoff. Next, patients were dichotomized according to the cutoffs, and all five proteins were significant predictors of survival. In details, poor survival was associated with a lower level of 4E-BP1_pT70, Chk1_pS345, and STAT5 alpha, and a higher level of Snail and PAI-1. Moreover, when tested in multivariate analysis with the 8th edition of the AJCC, three proteins (Chk1_p345, STAT5 alpha and PAI-1) were independent predictors of survival, whereas for 4E-BP1_pT70 and Snail Cox regression cannot be fitted because no event occurred in one of the two groups. Details were reported in Table 3. Finally, the five proteins were considered as a panel; a positive result was assigned whenever at least four out of five protein markers were above (or below) the selected cutoff. The panel was significantly associated with survival (*P* < 0.0001, Fig. 4), even independently from the AJCC staging, with an HR of 21.2 (95% CI 3.7–122.2, *P* = 0.0006).

**Table 3:** Testing of the best five proteins in discriminating patient’s vital status

Protein	AUC	Cutoff	Sensitivity	Specificity	PPV	NPV	Univariate *P*	Multivariate *P*	HR (95%CI)
4E-BP1_pT70	0.80	0.374	1	0.576	0.164	1	0.0002	NA^a^	NA^a^
Chk1_pS345	0.79	0.279	0.727	0.871	0.320	0.975	<0.0001	0.0079	9.4 (1.8–49.2)
Snail	0.77	−0.541	1	0.462	0.134	1	0.01	NA^a^	NA^a^
STAT5 alpha	0.77	1.160	0.818	0.713	0.188	0.979	0.01	0.0126	10.1 (1.6–62.5)
PAI-1	0.76	1.357	0.727	0.879	0.333	0.975	0.0001	0.0185	6.9 (1.4–34.0)
5-protein panel	0.89	4 out of 5 markers	0.818	0.954	0.600	0.984	<0.0001	0.0006	21.2 (3.7–122.2)

^a^For these cases the multivariate Cox regression cannot be fitted due to the lack of adverse events in one of the two groups obtained according to the cutoff

*AUC* area under the curve, *PPV* positive predictive value, *NPV* negative predictive value, *HR* hazard ratio, *CI* confidence interval, *NA* not available

## Discussion

The TCGA study on PTC provided the most comprehensive characterization of the molecular landscape of PTC [10]; however, the available follow-up period is short, especially in the context of WDTC. After the TCGA milestone, other studies have provided a comprehensive molecular characterization of PTC, even in light of the prognostic stratification [17]. For instance, Yoo and colleagues reported that advanced WDTC often harbor secondary mutations such as *TERT* promoter, *AKT1*, *PIK3CA*, and *EIF1AX*. In addition, some WDTC have an expression profile that is different from the three molecular subtypes of PTC (i.e., *BRAF*-like, *RAS*-like, and Non-*BRAF*-Non-*RAS*, NBNR). This fourth group closely resemble to anaplastic thyroid cancer (ATC) and was consequently named ATC-like. Herein, by using TCGA clinical-pathological and molecular data, we evaluated whether one or more molecular features could help in the patients’ risk stratification, also in the light of the AJCC staging system updating.

The 8th edition of the AJCC staging system produced the downstaging of a remarkable number of cases (Fig. 1 and Table 1) with the consequent neater separation of the risk of mortality among the four stages (Fig. 3). In the TCGA series, the proportion of deaths for each stage (i.e., 0.7, 6.2, 25, and 40% for stages I, II, III, and IV respectively) was lower but in line with the 10-year projection of expected survival [1, 2].

Besides the proved usefulness of gene mutations in diagnostics [18], they can be informative also for prognosis. In the absence of secondary mutations, *RAS*-driven lesions are generally low risk as confirmed by their association with the follicular variant and the absence of lymph node involvement in this series. Lesions positive only for *BRAF* mutations were generally associated with an intermediate risk, presence of central lymph node metastasis and minimal ETE. *TERT* promoter mutations deserve a separate discussion. *TERT* mutations were in fact the only type of mutations enriched in patient with persistence of disease and an unfavorable outcome. On one hand they can be highly informative when detected preoperatively because they should be a bell tolling since the presence of a high-risk lesion is very likely [9, 19]. On the other hand, their association with older age, aggressive pathological features and advanced stage can limit their usefulness after the pathological diagnosis and staging are made. In effect, the association of *TERT* promoter mutations with poor survival was already proved [9, 20, 21] and herein confirmed. *TERT* mutations are predictive of a poor outcome both alone [9, 20, 21] and in combination with *RAS* or *BRAF* alterations [9, 22]. Nevertheless, this should not be a solved issue, also because, with the downstaging occurring, a high number of *TERT* mutated cases were distributed into lower stages. For this reason, a longer follow-up and dedicated studies with a higher number of mutated cases are needed to understand whether *TERT* promoter mutations can help stratify patients at higher risk within low-stage diseases.

The majority of the other features analysed in the present study were predictive of poor survival including a higher MACIS score, a lower TDS, ATA high risk and the total number of nonsilent mutations. However, none of them was independent when tested in multivariate analysis with the latest edition of the AJCC staging. By analysing protein expression, we found that five proteins, namely 4E-BP1_pT70, Chk1_pS345, Snail, STAT5 alpha, and PAI-1, had a good performance in discriminating patient’s vital status (Table 3). Once chosen the best cutoff, all of them efficiently dichotomized patients with very different risk of death, and at least three of them were independent from the AJCC staging. By using these proteins as a five-marker panel, with a positive result rendered whenever at least four of them are above (or below) the selected cutoff, an effective stratification of patients was obtained (Fig. 4), and, most importantly, it was independent from the AJCC staging. The protein expression analysis is routinely and widely performed, mostly by immunohistochemistry (IHC); therefore, the five-protein panel could represent an appealing strategy to refine the risk stratification of patients. The cutoffs herein obtained are optimized for expression levels from rppa analysis, therefore they must be adjusted if another technique like IHC is used. The validation of these few markers as a new tool to predict the risk of poor outcome is warranted, especially in low-stage diseases.

Finally, the low rate of adverse events and the short follow-up period of this series could represent a limitation of the present study, thus, limiting the statistical power and requiring confirmation. Although patients with WDTC are not those with a higher risk of dying of thyroid cancer, adverse events occur also in this group of patients, albeit with a lower rate as herein observed. Moreover, the distribution of cases per stage and the proportion of deaths per each stage are in line with those reported and expected; thus, our findings are based on data that could reflect a real-life series of WDTC.

In conclusion, we provided some pieces of information to be used in the stratification of patients according to the risk of death. In addition, we proposed a five-protein-based stratification strongly predictive of patients’ survival, also independently from the AJCC staging.
